# Rodents as reservoirs of human pathogens in Bulgaria

**DOI:** 10.1186/1756-3305-7-S1-P12

**Published:** 2014-04-01

**Authors:** I Christova, I Trifonova, N Kalvatchev, H Dimitrov, V Mitkovska, T Gladnishka, V Ivanova

**Affiliations:** 1 Department of Microbiology, National Center of Infectious and Parasitic Diseases, Sofia, Bulgaria; 2 Department of Zoology, University of Plovdiv, Plovdiv, Bulgaria

## 

Small mammals are reservoirs of various human pathogens. The aim of this work was to investigate infections with human pathogens in rodents trapped in different regions of Bulgaria. A total of 284 rodents were investigated by PCR for detection of the flagellin gene of borreliae within *Borrelia burgdorferi *sensu lato complex, *ank*A gene of *Anaplasma phagocytophilum *and nucleoprotein gene of hantavirus Dobrava - conventional nested RT-PCR and Real Time PCR with TaqMan probe. *B. burgdorferi *was detected in 64/284 (22.5%) of the investigated rodents by PCR. Of them, 41 samples originated from *Apodemus flavicollis*, 20 from *A. agrarius*, and 3 from *A. sylvaticus*. Overall, 33 of the investigated 284 rodents were infected with *Anaplasma phagocytophilum *(11.6%) - 11 *Apodemus flavicollis *(infectivity rate 8.6% of the 128 investigated), 13 *Apodemus agrarius *(infectivity rate 13.5% of the 96 tested), and 1 *Apodemus sylvaticus *(infectivity rate 9% of the 11 tested). Hantavirus RNA was detected in 9 of the rodents. Only Dobrava-Belgrade virus but not Puumala or Saaremaa virus was detected. Almost all infected rodents were *A. flavicollis *(8/9 PCR-positive rodents). Rodents are important reservoirs of human pathogens. In this study, active infection in rodents was confirmed by detection of microorganism's genome. Remarkably, a high number of rodents from genus *Apodemus *were infected with borreliae. The high rate of detection of *A. phagocytophilum *in rodents from *A. agrarius *species suggested that this species might serve as major reservoir of human anaplasmosis in Bulgaria. Mainly infected with hantaviruses were *A. flavicollis *mice, known as reservoir of Dobrava hantavirus but hantavirus infections were detected also in *A. agrarius *mice. Medical authorities should be aware of the risk for humans.

